# Materia Medica, Pharmacy, Pharmacology, and Therapeutics

**Published:** 1893-03

**Authors:** 


*Materia Medica, Pharmacy, Pharmacology, and Therapeutics.* By W. HALE WHITE, M.D., F.R.C.P. Pp. vi., 614. London: J. & A. CHURCHILL. 1892.

In order to render complete his publications on Therapeutics, Dr. Hale White has brought out a text-book of Materia Medica, &c., in continuation of his work on General Therapeutics published a year or two ago. The book now under notice is moderate in size, not unduly ambitious, and with admirable succinctness gives account of the medicinal substances included in the British Pharmacopœia. It has the further advantage also of possessing, in a separate Appendix, descriptions of the chief non-official remedies now in use. This is as it should be, for even a student's knowledge of drugs must not be restricted to those enumerated in the B.P. While possessing perhaps no other special feature, the book is well adapted to students' use, and may be recommended as being abreast of the pharmacological and therapeutical knowledge of the day.

